# Insomnia among Medical and Paramedical Students in Jordan: Impact on Academic Performance

**DOI:** 10.1155/2019/7136906

**Published:** 2019-10-31

**Authors:** Mohammad Alqudah, Samar A. M. Balousha, Othman Al-Shboul, Ahmed Al-Dwairi, Mahmoud A. Alfaqih, Karem H. Alzoubi

**Affiliations:** ^1^Jordan University of Science and Technology, School of Medicine, Department of Physiology and Biochemistry, Irbid, Jordan; ^2^Jordan University of Science and Technology, School of Medicine, Department of Clinical Pharmacy, Irbid, Jordan

## Abstract

**Background:**

Insomnia is a problem that is common in all societies and age groups. However, its importance is increasing between students especially with the highly competitive and demanding environment surrounding them even after their graduation. In spite of the deep understanding of its health and social consequences, the frequency of insomnia among medical students in Jordan was not determined.

**Aim:**

To determine the prevalence of sleep disturbances among college students and to look for any association between sleep disturbances and students' academic achievement.

**Methods:**

This is a cross-sectional self-administered questionnaire-based study. The participants were college students of the medical and paramedical specialties. Insomnia Severity Index (ISI) was used and the academic performance was assessed using students' Cumulative Grade Point Average (CGPA).

**Results:**

There were 977 responses. Prevalence of clinical insomnia was 26.0%. Students who self-reported good sleep quality had significantly lower ISI scores compared with those who self-reported bad quality of sleep. Students who slept >7 hours had significantly less ISI scores than students who slept <6 hours. Students who had a CGPA more than or equal to 3 had significantly lower ISI scores compared with those who had a CGPA less than 2.5. Self-reported sleep quality was associated with the CGPA.

**Conclusion:**

A high prevalence of insomnia was found in this group of students. Academic performance was significantly associated with ISI scores and self-reported sleep quality. These results might be useful for future research into the development of interventional strategies to help students get enough sleep quality and quantity.

## 1. Introduction

Representing one-third of our life span, sleeping considered an important biological and behavioral constituent of human physiology [1]. As such, metabolic and energy re-boosting for proper brain function during the daily waking hours can occur during our sleep [2, 3]. In agreement with that, sleep deprivation can directly lead to hallucinations and delusional behaviors [3] and can indirectly predispose to multiple systemic diseases [4, 5] which cumulatively can affect the humans' quality of life. Motor-vehicle accidents, decreased work productivity [6], and psychological illnesses like anxiety and depression [7, 8] can be associated with or a consequence of sleep deprivation.

The majority of adults will normally need between 7.5 and 8.5 hours of sleep per day [9]. However, the sleeping pattern may not be similar in all of them [10]. The lifestyle of each person, the age, the physical and psychological health status, and many other factors can affect the mode of sleep for each person. Mainly, the non-rapid eye movement (NREM), and specially Stage 1 of it, is the main sleeping period which can be affected and prolonged during sleeping disturbances [2, 9].

Insomnia is the main sleep disturbance that regular people can recognize and complaint about [11], and it can be either difficulty in initiating the sleep process or maintaining it more than 7 hours [2]. It is very common between adults, and some reports indicated that approximately 30% of them can suffer from insomnia at any time [11]. In western countries including the USA, insomnia prevalence was between 10 and 48% of the general populations [12, 13]. Thereafter, increased awareness about this problem was evidenced in the last few years [9].

Among university students, sleep deprivation may be significantly associated with memory reduction and reduced learning abilities which ultimately can lead to reduce their academic achievements [14–16]. This evidenced with medical students in particular [17]. The prevalence of insomnia among those students can range from 9.5 to 27% based on recent studies [18, 19]. Stress, the need to accomplish distinguished achievements, physiological sleep problems during adolescence, and annoying lifestyle of the universities' dorms are among many factors that can affect the mode of sleep for this group of students [20].

Several studies had reported the prevalence of sleep disorders among college students in different countries of the world. However, to our knowledge, no studies have examined the prevalence of sleep disturbances in college students in Jordan and the relationship between that and their academic performance.

It is very important to detect sleep disorder in such a group. This study will provide a better understanding of sleep problems burden in college students so that we can prevent future health problems, reconsider heavy college schedules, and improve sleeping habits for better academic achievement in this population.

The objective of the current study is to determine the prevalence of sleep disturbances among medical and paramedical students and to look for any association between sleep disorder and college students' academic achievements.

### 1.1. Participants and Methodology

This was a cross-sectional questionnaire-based, observational study carried out during the period from November to December 2018 among students enrolled at the Jordan University of Science and Technology, Irbid, Jordan. The cross-sectional study design was selected because of its capacity to answer the goals of the study in a convenient manner in terms of time and resources. The protocol of the study was approved by the Institutional Review Board (IRB) of the Jordan University of Science and Technology. The study population consisted of students enrolled from 5 different colleges; Medicine and Surgery, Dentistry, Nursing, Pharmacy and Pharm. D, and Applied Medical Sciences at the Jordan University of Science and Technology, Irbid, Jordan.

The researcher approached the students at open areas in the medical campus buildings, in classrooms during the rest period between classes, and in the food court. The questionnaire was distributed by the researcher. Students completed it personally, and the researcher was present for any inquiries from the participants. Each member was provided with a full clarification of the study and how to finish the survey. Students were informed that being enrolled in the study is voluntary. Confidentiality was maintained as no names and addresses of participants were required.

### 1.2. Study Questionnaire

A self-administered questionnaire was developed based on literature review and Insomnia Severity Index (ISI). Academic performance was assessed using students' Cumulative Grade Point Average (CGPA).

The study questionnaire was aimed to identify insomnia prevalence and its severity and other sleep-related variables among medical and paramedical students. The students were asked to limit their responses to incidents occurred during the past month.

### 1.3. ISI

Insomnia Severity Index (ISI) is a seven-item, self-report questionnaire that measures the person's perception of his/her insomnia during the past month. It consists of 7 questions assessing the severity of sleep initiation, sleep maintenance, early morning awakening, sleep dissatisfaction, sleep interference with daily activities, noticeability of sleep problems by others, and distress caused by the sleep problems. Each item is scaled on a 5-point Likert scale from 0 to 4 to yield a total score ranging from 0 to 28. Interpretation of the results is as follows: absence of insomnia (0–7); subthreshold insomnia (8–14); moderate insomnia (15–21); and severe insomnia (22–28) [21–23]. Numerous studies proved that ISI is a valid and reliable instrument [24]. The internal consistency was established by using Cronbach's alpha test. Cronbach's alpha value for ISI was 0.75. The ISI questionnaire was in English language.

In this study, the Statistical Package for Social Sciences (SPSS) software version 22.0 was used for data entry, tabulation, and analysis. Descriptive and inferential statistics were used to analyze the data in this study. Pearson's chi-square was used to test the association between categorical variables. Independent samples *t*-test and one-way analysis of variance (ANOVA) test were applied for the comparison of ISI scores between and among categorical groups. If statistical significance was observed, Tukey HSD post hoc tests were applied to understand pairwise group differences. Linear regression was performed to predict the ESS (dependent) variable from the presence of insomnia, actual hours of sleeping, and age (independent) variables.

## 2. Results

### 2.1. Demographic Characteristics of the Participants

A total of 977 participants were recruited from medical and paramedical students, of whom 361 (36.9%) students were males and 616 (63.1%) were females, which is in keeping with the gender distribution of the colleges. The mean age of participants was 20.9 ± 2.2 years. Five colleges were included from which 152 (15.6%) students were from Nursing and Midwifery college, 154 (15.8%) from Pharmacy and Pharm. D college, 159 (16.3%) from Dentistry College, 212 (21.7%) from Applied Medical Sciences College, and 299 (30.6%) from Medicine and Surgery College.

According to the academic level, 156 (16.1%) students were from the 1^st^ year, 218 (22.5%) from the 2^nd^ year, 150 (15.5%) from the 3^rd^ year, 218 (22.5%) from the 4^th^ year, and 228 (23.5%) students from the 5^th^ and 6^th^ years.

Using sleeping pills during the past 30 days, at least less than once a week, was reported by 22.4% of the students. Further details about and other variables are shown in Table 1.

### 2.2. Insomnia Prevalence and Severity (ISI)

The presence of insomnia was evaluated according to the Insomnia Severity Index (ISI) scale. Prevalence of clinical insomnia was 26.0%. In addition, the prevalence of subthreshold insomnia was 49.9%. The mean scores of the sample ISI were 11.2 ± 5.2 as shown in Table 2. For association, seven were used as the cutoff for the presence of insomnia based on expert opinion. Neither gender nor specialization was significantly associated with ISI scores. However, a statistically significant relationship was evident with the academic level, sleep quality, and sleeping hours (*p* < 0.05) as demonstrated in Table 3. *Academic level*: the students in the 2^nd^ year had a significantly higher mean of ISI compared with the 6^th^ year students. Also, the students in the 3^rd^ year had a significantly higher mean of ISI scores compared with the 6^th^ year students. *Sleep quality*: students who had a good self-reported sleep quality had a significantly lower mean of ISI scores compared with those who reported bad quality of sleep. *Sleeping hours*: students who slept more than 7 hours had a mean of ISI score significantly less than those who slept less than 5 hours and 5–5.9 hours. Also, students who slept less than 5 hours had a mean of ISI score significantly higher than those who slept 6–6.9 hours.

### 2.3. Specialization and Other Sleep-Related Variables

In Table 4, the data show that there was a statistically significant association between specialization and other sleep-related variables, namely, presence of insomnia and sleeping hours at night. The percentage of insomnia was highest among Pharmacy and Pharm. D students and lowest among Medicine and Surgery students, Pharmacy and Pharm. D (81.8%), Dentistry (79.2%), Applied Medical Sciences (79.1%), Nursing (973%), and Medicine and Surgery (70.2%).

Moreover, students who slept less than five hours at night were highest from college of Dentistry (23.9%). And those who slept more than 7 hours at night were highest from college of Nursing and Midwifery (36.2%).

Comparing students from the Pharmacy and Pharm. D college with the other students, we found that students from this college were using sleeping pills more than students from the other colleges with a percentage of 29.9% (*X*^2^ = 5.8, *p*=0.02).

### 2.4. Cumulative Grade Point Average (CGPA)

Table 4 shows that the students who had a CGPA of 3.5 and above had a significantly lower means of ISI scores (10.79, *p*=0.003) compared with those who had a CGPA less than 2.5 (13.69, *p*=0.003). Also, students who had a CGPA of 3–3.49 had a significantly lower mean of ISI scores (11.6, *p*=0.003) compared with those who had a CGPA less than 2.5.

**Figure 1:**
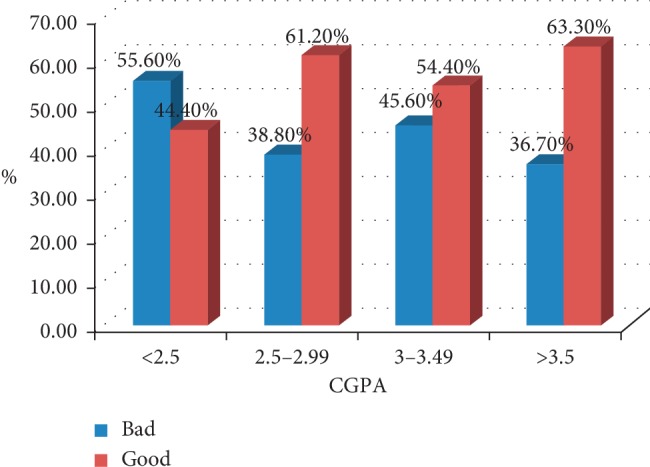
The association between self-reported sleep quality and CGPA. Self-reported sleep was assessed as Good and Bad, and the percentage was plotted against the CGPA. The overall diagram concludes that CGPA is dependent on the sleep quality, *p*=0.02.

Moreover, CGPA is significantly associated with sleep quality, a high percentage of self-reported good sleep quality was found in students who had a CGPA of 3.5 and above (63.3%, *p*=0.02) and a high percentage of self-reported bad sleep quality was found in students who had a CGPA of less than 2.5 (55.6% *p*=0.02) Figure 1.

## 3. Discussion

Approximation of the prevalence of insomnia relies upon how we define insomnia and more importantly which population is studied. A general agreement has been created from population-based studies that around 30% of adults recruited from different nations report at least one of the symptoms of insomnia: difficulty initiating sleep, difficulty maintaining sleep, waking up too early, and sometimes, nonrestorative or low quality of sleep [25]. A systematic review on the prevalence of insomnia among college students performed in December 2015 showed that the prevalence of insomnia in the general population was 7.4%; however, the prevalence among university students ranged from 9.4% to 38.2% and the weighted mean prevalence is 18.5% which is significantly higher than in the general population [26]. A cross-sectional study carried out in Ethiopia among university students collected from nine colleges demonstrated that the prevalence of insomnia was 61.6% [27].

In this study, insomnia was defined as scoring more than 7 in ISI scores. The result of this study revealed that prevalence of subthreshold and clinical insomnia among medical and paramedical students is 75.9% of which 49.9% is subthreshold and 26.0% is clinical, which is in congruence with cross-sectional studies report in Hong Kong, 68.8% [28] and in Germany [29]. However, it is considerably higher than the prevalence in Nigeria [30] and Ethiopia [27, 31]. This difference may be the result of different ways of defining insomnia and divergence of sociodemographic characteristics [32]. A cross-sectional study carried out in Lebanon [33] which used the same index (ISI) as ours for defining insomnia showed a prevalence of subthreshold and clinical insomnia in university students of 72.1% which is comparable with our result, yet the prevalence of clinical insomnia in the same study was only 10.6%, which is considerably lower than our result that showed 25.9% of students suffering from clinical insomnia. In the current study, neither gender nor specialization was significantly associated with ISI scores, which is parallel to what Choueiry [33] found. In addition, our result revealed a statistically significant association between ISI scores and academic year with higher mean found in the earlier academic years, namely, 2^nd^ and 3^rd^ years, this might indicate that students in the 2^nd^ and 3^rd^ academic years are under more sleep disturbances and possibly more pressure. Choueiry showed more frequent clinical insomnia in 1^st^ year students [33].

Our results showed that only 26.1% of students get more than 7 hours of sleep at night, which is approximately equal to the average amount of hours required for young adults [34]. Forty-two percent of students reported getting less than 6 hours of sleep at night. A study conducted in a large population of college students showed 25% of students get less than 6.5 hours of sleep at night and that 29.4% have ≥8 hours of actual sleep per night [35].

The relationship between academic performance and sleep problems have been demonstrated in multiple studies. A cross-sectional study reported an association between poor sleep qualities in terms of abnormal PSQI and lower academic performance [36]. Other studies showed similar results [37, 38]. Our results were not different from other studies in this regard as it demonstrated that self-reported sleep quality was dependent on the CGPA. The opposite way, a study among Palestinian students demonstrated no association between sleep quality and academic achievement [39].

In this study, Pharmacy students showed a high percentage of insomnia 81.8% followed by dentistry students 79.2%. Moreover, Pharmacy students who slept less than 5 hours or 5–5.9 hours were about 50% of the students, which is consistent with other findings [40, 41]. The comparison between specializations and insomnia is underinvestigated in the literature. However, our finding that Pharmacy students have high insomnia rate needs further studies to investigate its reasons and consequences. Furthermore, this observation warns the faculty members and the administrative people in the school of Pharmacy to increase the awareness among their students of good sleep hygiene.

The present study showed a significant relationship was evident between CGPA and ISI scores. A study by Haile reported no relationship between insomniac students and their CGPA (28).

## 4. Conclusion and Recommendation

In conclusion, our study reveals a high prevalence of insomnia among medical and paramedical students. The analysis of the relationship between self-reported sleep quality and academic achievement shows a significant relationship. Also, a significant association was found between ISI and CGPA. Thus, students should be educated about the importance of good sleep hygiene and quality. In future, there is a need to investigate the causes of the high prevalence of sleep disturbances among college students in Jordan such as poor sleep hygiene and the use of technology, as well as to study the impact of sleep problems on other aspects such as health status especially mental health.

We strongly recommend the implementation of workshops to increase awareness of the importance of good sleep quality and to apply insomnia and sleep quality screening programs for students. Moreover, application of decreased course schedules is recommended.

## Figures and Tables

**Table 1:** Demographic characteristics and other variables of the study population (*n* = 986).

No.	Variable	Statistics^*∗*^
1	Age	20.9 ± 2.2
2	Gender	
	Male	361 (36.9%)
	Female	616 (63.1%)
3	College	
	Nursing and Midwifery	152 (15.6%)
	Pharmacy and Pharm. D	154 (15.8%)
	Dentistry	159 (16.3%)
	Applied Medical Sciences	212 (21.7%)
	Medicine and Surgery	299 (30.6%)
4	Academic year+	
	1^st^	156 (16.1%)
	2^nd^	218 (22.5%)
	3^rd^	150 (15.5%)
	4^th^	218 (22.5%)
	5^th^	120 (12.4%)
	6^th^	108 (11.1%)
5	Medical problem	
	Yes	109 (11.2%)
	No	868 (88.8%)
6	Sleeping hours at night	
	<5	155 (15.9%)
	5–5.9	256 (26.3%)
	6–6.9	310 (31.8%)
	>7	254 (26.1%)
7	Frequency of using sleeping pills (prescribed or over the counter)	
	Not during the past month	757 (77.6%)
	Less than once a week	103 (10.6%)
	Once or twice a week	73 (7.5%)
	Three or more times a week	43 (4.4%)
8	Sleep quality	
	Good	570 (58.8%)
	Bad	399(41.2%)
9	Academic scores (CGPA)	
	Below 2.5	45 (4.9%)
	2.5–2.99	202 (26.7%)
	3–3.49	321 (34.7%)
	3.5 and above	358 (38.7%)

^*∗*^Statistics are expressed as mean ± SD for continuous variables and as a frequency for categorical variables. Numbers (proportions) of participants endorsing each response (*N* = 977). Variables are gender, colleges, level, sleeping hours, frequency of using sleeping pills, sleep quality, and CGPA. Students from the 6^th^ academic year include only students from the Medicine and Pharm. D colleges.

**Table 2:** Insomnia, ISI scores, and interpretation.

No.	Variable	Statistics^*∗*^
1	Insomnia	
	Present	741 (75.9%)
	Absent	235 (24.1%)
2	ISI scores	11.2 ± 5.2
3	ISI interpretation	
	Absence of insomnia (0–7)	235 (24.1%)
	Subthreshold insomnia (8–14)	487 (49.9%)
	Clinical insomnia, moderate to severe (15–28)	254 (26.0%)

^*∗*^Statistics are expressed as mean ± SD for continuous variables and as a frequency for categorical variables. Numbers (proportions) of participants endorsing each response (*N* = 977). Pearson's chi-square was used to test the association between categorical variables, *p* value ≤ 0.05 was considered significant.

**Table 3:** Total ISI scores with students characteristics.

	Total ISI score		
	Mean ± SD	Median (min–max)	*p*
Gender			
Male	11.1 ± 5.5	11.0 (0–28)	0.74
Female	11.2 ± 4.9	11.0 (0–27)	
Academic year			
1^st^	10.3 ± 5.1	10.0 (0–25)	<0.05
2^nd^	12.3 ± 5.1	12.0 (0–27)	
3^rd^	11.9 ± 4.6	12.0 (0–25)	
4^th^	11.1 ± 5.4	11.0 (0–28)	
5^th^	10.7 ± 4.2	11.0 (1–20)	
6^th^	10.0 ± 5.8	10.0 (0–25)	
College			
Nursing	10.7 ± 4.9	10.5 (0–26)	0.10
Pharmacy and Pharm. D	12.1 ± 4.9	12.0 (0–23)	
Dentistry	11.2 ± 5.0	11.0 (0–27)	
Applied medical science	11.3 ± 5.1	11.0 (0–27)	
Medicine	10.8 ± 5.4	11.0 (1–28)	
Presence of insomnia			
Insomnia	13.3 ± 3.9	13.0 (8–28)	<0.05
No insomnia	4.6 ± 2.0	5.0 (0–7)	
Sleep quality			
Good	9.1 ± 4.5	9.0 (0–22)	<0.001
Bad	14.4 ± 4.6	14.0 (7–28)	
Sleeping hours			
>7	10.0 ± 5.3	10.0 (0–25)	<0.05
6–6.9	10.0 ± 4.6	10.0 (0–24)	
5–5.9	12.2 ± 4.8	12.0 (0–24)	
<5	14.0 ± 5.0	14.0 (4–28)	
CGPA			
>3.5	10.79 ± 5.1	11.0 (0–26)	0.003
3–3.49	11.31 ± 4.7	11.0 (0–23)	
2.5–2.99	11.60 ± 5.5	12.0 (0–27)	
<2.5	13.69 ± 5.9	14.0 (0–28)	

Table shows the results of independent samples *t*-test and ANOVA of the total ISI scores with student's characteristics; *p* value of 0.05 is considered significant.

**Table 4:** Specialization and sleep-related variables.

	Nursing (%)	Pharmacy (%)	Dentistry (%)	Applied Medical Sciences (%)	Medicine and Surgery (%)	Chi-square (%)	*p* value
Presence of insomnia							
Insomnia	73.0	**81.8**	79.2	79.1	70.2	11.1	<0.05
No insomnia	27.0	18.2	20.8	20.9	**29.8**		
Total	100	100	100	100	100		
Sleeping hours at night							
<5	14.5	19.5	**23.9**	12.8	12.8	38.6	<0.001
5–5.9	21.1	27.9	25.8	26.5	28.2		
6–6.9	28.2	34.4	31.4	26.1	36.5		
>7	**36.2**	18.2	18.9	34.6	22.5		
Total	100	100	100	100	100		
Sleep quality							
Good	**62.5**	59.1	55.8	61.9	56.1	3.2	0.53
Bad	37.5	40.9	**44.2**	38.1	43.9		
Total	100	100	100	100	100		
Using sleeping pills							
No	80.3	70.1	**83.0**	75.4	78.6	9.01	0.06
Yes	19.7	**29.9**	17.0	24.6	21.4		
Total	100	100	100	100	100		

Table illustrates the results of chi-square between the specializations (in columns) and other categorical variables (in rows). A statistically significant association was evident with the presence of insomnia and sleeping hours. The highest percentages are bolded, *p* value ≤ 0.05 is considered significant.

## Data Availability

The data used to support the findings of this study are included within the article.

## Additional Points

*Limitations*. Due to the fact that students self-reported their CGPA, it is unsure whether accurate scores were given. Also, the causes of sleep problems were not addressed in the present study. Because this study was conducted in only one university, results may not be generalized to students at other universities. Moreover, the responses of the students were limited to sleep behaviors and incidents that occurred in one month before conducting the survey. Therefore, the correlation between academic performance and sleeping behavior may not be very accurate and need to be interpreted with caution.
